# Diffusion Tensor Imaging of the Kidney: Design and Evaluation of a Reliable Processing Pipeline

**DOI:** 10.1038/s41598-019-49170-5

**Published:** 2019-09-04

**Authors:** Pasquale Borrelli, Carlo Cavaliere, Luca Basso, Andrea Soricelli, Marco Salvatore, Marco Aiello

**Affiliations:** 1 IRCCS SDN, Diagnostic Imaging, Naples, 80143 Italy; 2 University of Naples Parthenope, Department of Motor Sciences and Healthiness, Naples, 80133 Italy

**Keywords:** Kidney, Biomedical engineering

## Abstract

Diffusion tensor imaging (DTI) is particularly suitable for kidney studies due to tubules, collector ducts and blood vessels in the medulla that produce spatially restricted diffusion of water molecules, thus reflecting the high grade of anisotropy detectable by DTI. Kidney DTI is still a challenging technique where the off-resonance susceptibility artefacts and subject motion can severely affect the reproducibility of results. The aim of this study is to design a reliable processing pipeline by assessing different image processing approaches in terms of reproducibility and image artefacts correction. The results of four different processing pipelines (*eddy*: correction of eddy-currents and motion between DTI volume; *eddy*-*s2v*: *eddy* and within DTI volume motion correction; *topup*: *eddy* and geometric distortion correction; *topup*-*s2v*: *topup* and within DTI volume motion correction) are compared in terms of reproducibility by test-retest analysis in 14 healthy subjects. Within-subject coefficient of variation (wsCV) and intra-class correlation coefficient (ICC) are measured to assess the reproducibility and Dice similarity index is evaluated for the spatial alignment between DTI and anatomical images. *Topup*-*s2v* pipeline provides highest reproducibility (wsCV = 0.053, ICC = 0.814) and best correction of image distortion (Dice = 0.83). This study definitely provides a recipe for data processing, enabling for a clinical suitability of kidney DTI.

## Introduction

Magnetic resonance (MR) diffusion tensor imaging (DTI) is a powerful technique that allows *in vivo* estimation of the water random movement. It is based on diffusion weighted imaging (DWI), a non-invasive technique that provides useful information on tissue diffusion properties as well as microstructural changes in different pathologies^1–3^. In addition to the diffusion estimation revealed by DWI, DTI allows the analysis of anisotropic properties of the tissues by returning the preferential water diffusion directions, quantitatively represented by the fractional anisotropy (FA). Due to the restricted water molecular mobility in organized tissue structures, DTI has been used to provide information on tissue organization as well as microstructural integrity^4,5^.

Mainly used in neuroimaging studies for the characterization of brain white matter tracts^6^, DTI has been recently applied to different intra-abdominal organs^7^ including prostate^8–10^, liver^11,12^ and kidney^13,14^. DTI is particularly suitable for kidney studies due to well-defined structural organization. Indeed, tubules, collector ducts and blood vessels in the medulla produce spatially restricted diffusion of water molecules, thus reflecting the high grade of anisotropy detectable with DTI. Since the first DTI analysis of the kidney described by Ries *et al*.^13^ in which the anisotropy differences revealed by DTI in the medulla with respect to the cortex were proven, further studies demonstrated that FA values of the medulla in healthy kidneys are higher compared to that of the cortex^14–16^. Moreover, since DTI does not require any contrast agent administration, it is suitable for the imaging of subjects with kidney dysfunction, for which the use of contrast agents is highly contraindicated. In this scenario, recent studies demonstrated the ability of DTI to detect renal damage in chronic kidney diseases, glomerulonephritis and renal lesions^17–20^. DTI was also applied in subjects after renal allograft to study the differences of diffusion parameters between transplanted and healthy kidney and the correlation with clinical parameters as the estimated glomerular filtration rate^21,22^.

DTI is typically obtained by acquiring a set of spin echo echo-planar imaging (EPI) sequences with different diffusion directions and b-values. Due to the rapid switching of diffusion encoding gradients and the low bandwidth in phase encoding direction, it is well known that DTI images obtained with EPI suffer from off-resonance induced distortions as eddy-currents and susceptibility-induced geometric distortions^23^. Moreover, the need to obtain DTI images with adequate spatial and angular resolution results in prolonged scan time, thus producing increased subject movement both between and within DTI volumes during the acquisition. Therefore, in this context ad hoc image processing methodologies able to correct or mitigate image artefacts are recommended.

Recent advances in brain DTI processing methods demonstrated robust results by reducing the effects of image artefacts. In particular, different strategies were developed to deal with eddy-currents distortions as well as subject movement^24,25^. Moreover, several methods have been proposed to correct susceptibility-induced geometric distortion by using an additional DTI image acquired with reversed phase encoding direction^26,27^ or by retrospective deformable registration procedures^28,29^. More recently, a novel methodology correcting the motion occurring both between (volume-to-volume) and within (slice-to-volume) each DTI volume was proposed^30^. However, as the aforementioned image processing methods have mainly been developed for processing brain DTI, their use was poorly or never investigated in kidney DTI where the image mis-registration due to subject motion and the geometric distortion artefacts are extremely detrimental. In this scenario, in order to assess the clinical utility of kidney DTI, the reliability of the results derived from DTI could be demonstrated by the standardization of both acquisition protocols and image processing pipelines^31^.

In this study, different image processing steps are evaluated to provide an optimal image processing protocol for kidney DTI. In this setting, in order to address the aforementioned image artefacts affecting kidney DTI, different strategies, both in acquisition and processing step, which tackle the eddy-currents, susceptibility-induced distortion and motion both between and within DTI volumes, are applied. Moreover, a test-retest reproducibility analysis is conducted for each tested processing pipeline in order to assess the reproducibility of the results. In literature several studies were based on reliability and repeatability analysis in both renal DWI and DTI^14,32–34^ of healthy subjects, thus demonstrating the repeatability of DTI parametric maps with a typical processing pipeline. However, to the best of our knowledge, this is the first study aimed to assess the effects of different processing pipelines to the reproducibility of the kidney DTI. Moreover, this is the first study where the slice-to-volume motion correction is included as preprocessing step in kidney DTI.

## Methods

The study was approved by Local Ethics Committee (IRCCS Pascale) with protocol number 17/17. Written informed consent was obtained for all participants and all the procedures adopted in this study were performed in accordance with the approved guidelines and regulations. Between May and September 2018, 14 healthy volunteers (age range: 22–37 years, mean ± standard deviation: 28 ± 4 years, median: 27 years, 4 women) with no history of kidney pathologies, vascular diseases, diabetes, hypertension and no contraindications for MR exam were enrolled in this study.

### MR examination

MR exam was performed for all participants using a 3T scanner (Biograph mMR; Siemens, Erlangen, Germany) with a 4-channel phased-array body coil. Since it was already demonstrated that the DTI parametric map values are not influenced by subject fasting or hydration status^32,35^, the MR exam was performed without any specific subject preparation. Anatomical image, within a field of view comprising both left and right kidneys, was acquired in breath-hold using an oblique-coronal T2-weighted half-Fourier acquisition single-shot turbo spin echo (HASTE) with following parameters: TR/TE, 1400/91 ms; slice thickness 5 mm; field of view, 380 × 380 mm^2^; matrix, 320 × 320; pixel size, 1.188 × 1.188 mm^2^; slices, 36; parallel imaging accelerator factor, 2. DTI images were acquired with a respiratory-triggered oblique-coronal fat-saturated twice-refocused EPI sequence with the following parameters: b-values, 0 and 500 s/mm^2^; number of images at *b* = 0 s/mm^2^, 6; diffusion directions, 6; TR/TE, 1800/74 ms; averages, 3; slices, 23; slice thickness, 3 mm; field of view, 319 × 319 mm^2^; matrix, 152 × 152; pixel size, 2.1 × 2.1 mm^2^; parallel imaging acceleration factor, 2. Depending on the individual respiratory cycle, acquisition time varied from 5 to 6 min. Two sets of DTI data were acquired with the phase-encoding direction reversed (left to right and right to left) to enable the geometric distortion correction resulting from EPI acquisition^26^. In order to quantify the reproducibility of kidney DTI, the DTI sequence was repeated in the same MR session without moving the subject into the scanner, thus obtaining two sets of DTI images for each participant.

### Image processing

The image quality for each participant was visually checked by an expert radiologist with more than 5 years of experience in abdominal imaging. Moreover, as additional quality check, an automated image quality control^36^ was applied to the DTI images. In particular, the mean absolute (i.e. with a reference volume) and relative (i.e. with the previous volume) motion between different volumes of each acquired DTI were calculated by means of averaging translation and rotation parameters across all voxels.

Before the evaluation of the parametric maps derived from DTI, four different data processing pipelines (summarized in Supplementary Table S1) were designed:

- correction of eddy-currents and volume-to-volume movement (indicated as *eddy*);
- correction of eddy-currents, volume-to-volume and slice-to-volume movement (indicated as *eddy-s2v*);
- correction of eddy-currents, volume-to-volume movement and susceptibility-induced distortion correction (indicated as *topup*);
- correction of eddy-currents, volume-to-volume and slice-to-volume movement and susceptibility-induced distortion correction (indicated as *topup-s2v*).

All the processing pipelines were applied by using FMRIB-FSL (v. 6.0.0) library^37^. In particular, eddy-currents distortions and volume-to-volume movement were corrected with FSL eddy function^24^. Moreover, the susceptibility-induced geometric distortions were corrected by using the additional DTI with reversed phase-encoding direction^26^. Finally, the slice-to-volume movement was corrected by the registration method presented in Andersson *et al*.^30^.

For each processing pipelines, FA, mean diffusivity (MD), radial diffusivity (RD) and axial diffusivity (AD) parametric maps were evaluated from the eigenvalues of the diffusion tensor by means of MRtrix3 software toolbox^38,39^ (available at http://www.mrtrix.org). Moreover, a tensor-based deterministic approach^40^ was used to whole-kidney tractography reconstruction with the following parameter: FA threshold of 0.1 and angle threshold of 60°. Tractography reconstructions were visually inspected for both anatomical and color-coding consistency.

Both parametric maps and tractography were evaluated also from DTI data without any image processing procedure, thus obtaining a total of five different sets of images for each participant. Finally, the mean between the *b* = 0 s/mm^2^ images, indicated as b0-image, was evaluated for each processing pipeline.

### Test-retest reproducibility

Regions of interest (ROIs) were annotated with the open source software ITK-SNAP v3.6.0^41^. A coronal slice in the central kidney section closest to the renal hilum was selected for the segmentation procedure. Three kidney sections corresponding to upper, middle and inferior pole were selected for ROI analysis. For each section, two circular ROIs of 58 mm^2^ were manually drawn in the renal cortex and medulla for both kidneys thus obtaining a total of twelve ROIs. The combination of b0-image and FA map was used to accurately define cortex and medulla and to avoid renal pelvis, vessels and calyces (see Supplementary Fig. S1). The ROI placement was performed on the test set and each ROI was copied to the corresponding retest dataset. Due to spatial misalignment between images obtained with different processing protocols, the ROI analysis was separately performed for every tested processing pipelines. For each set of parametric maps (FA, MD, RD, and AD), the segmented ROIs were overlaid on the DTI maps and mean and standard deviation were evaluated for both cortical and medullary regions. Moreover, mean and standard deviation of cortical and medullary values were separately extracted from left and right kidney.

A paired t-test was performed in order to assess the statistical differences between cortical and medullary ROI mean values. Moreover, a paired t-test was also used to statistically estimate the difference between values from left and right kidney. The statistical significance was assumed as p < 0.05. The test-retest reliability was quantitatively assessed both for cortical and medullary ROI mean values via within-subject coefficient of variation^42^ (wsCV) and intra-class correlation coefficient^43^ (ICC). The wsCV was evaluated by the ratio between the within-subject standard deviation and the mean value of the entire population. Values of wsCV < 0.1 reflected low variability between measurements, >0.1 and <0.2 represented moderate variability and >0.2 were considered for highly variable measurements^44^. ICC (two-ways random effect model, absolute agreement) was evaluated as:

1 $$ICC=\frac{BMS-WMS}{BMS+(k-1)WMS},$$

where *BMS* and *WMS* respectively are the between- and within-subject mean squares and *k* represents the number of repeated measurements. For the ICC parameter, a value >0.7 was considered for highly reliable measurements^44^.

### Geometric distortion correction assessment

Both kidneys were manually segmented on the HASTE image. The segmentation procedure consisted in generating 2D mask for each slice containing the kidney parenchima in the coronal plane thus composing a 3D binary mask. Border pixels isointense respect to the kidney parenchima were included during the segmentation process to avoid bias due to partial volume effects. In order to evaluate the results of geometric distortion correction routine, the same segmentation procedure was applied to the b0-image obtained from both DTI without processing and with *topup-s2v* pipeline. The binary kidney 3D masks obtained from b0-image were then resliced to the HASTE space in order to quantitatively compare the spatial overlap.

Dice similarity coefficient^45^ was used to quantitatively compare the 3D masks obtained from HASTE and b0-images. In particular, the Dice index was calculated as:

2 $$Dice=2\frac{(|A\cap B|)}{(|A|+|B|)},$$

where *A* and *B* correspond to the mask from HASTE and b0-image, respectively.

## Results

**Figure 1:**
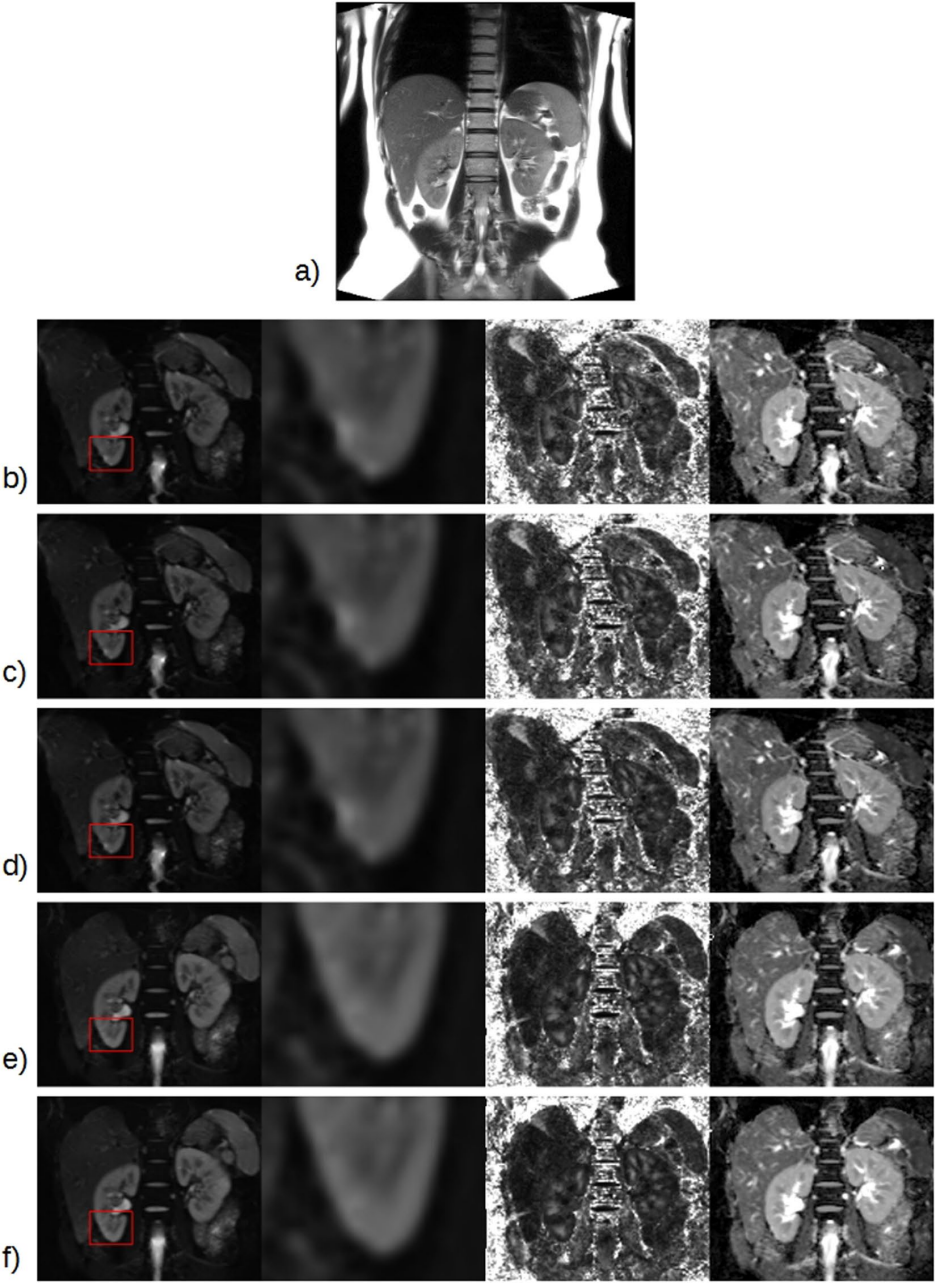
Acquired and processed images of a 28-year-old female volunteer. (**a**) HASTE image; (**b**–**f**) b0-image, an enlargement of b0-image and parametric maps (from left to right: FA, MD) obtained with different image processing pipelines ((**b**) without processing; (**c**) *eddy*; (**d**) *eddy-s2v*; (**e**) *topup*; (**f**) *topup-s2v*). A kidney region (corresponding to the red box in b0-image) was zoomed to highlight the differences of the results between the processing pipelines.

**Figure 2:**
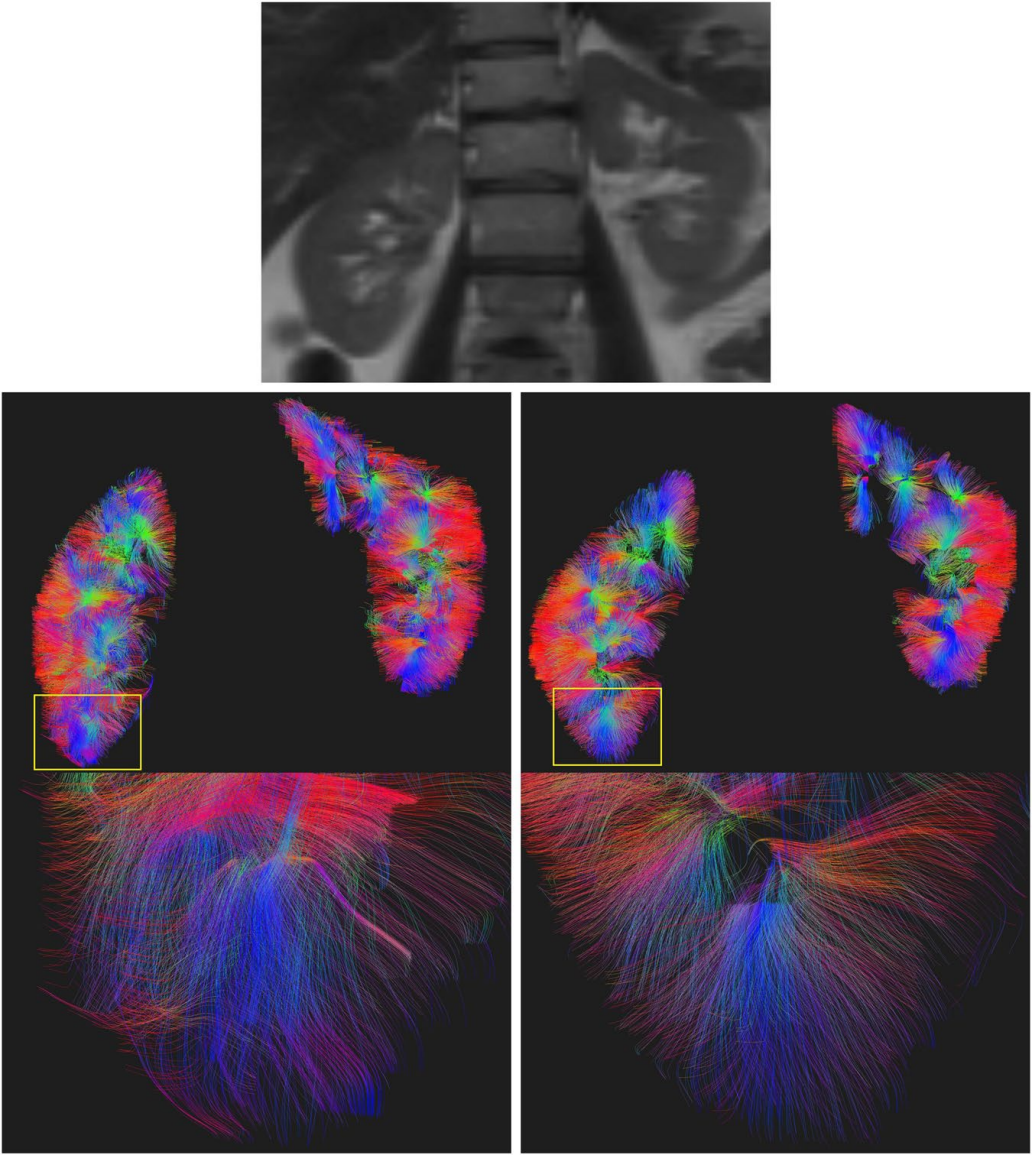
HASTE image (upper) and tractography reconstructions from DTI without processing (bottom left) and with *topup-s2v* pipeline (bottom right) of a 25-year-old female volunteer. A region of the tractography was zoomed to highlight the differences of the results between the processing pipelines.

All participants in this study completed the MR exam and no images were discarded after the preliminary visual inspection. The image quality is also confirmed with the control report (Supplementary Fig. S2) where both absolute and relative subject movement was always <1.5 mm. An example of HASTE and DTI-derived parametric maps is shown in Fig. 1. Moreover, an example of the tractography reconstructions from DTI without processing and from *topup-s2v* pipeline is reported in Fig. 2.

### Test-retest reproducibility

**Figure 3:**
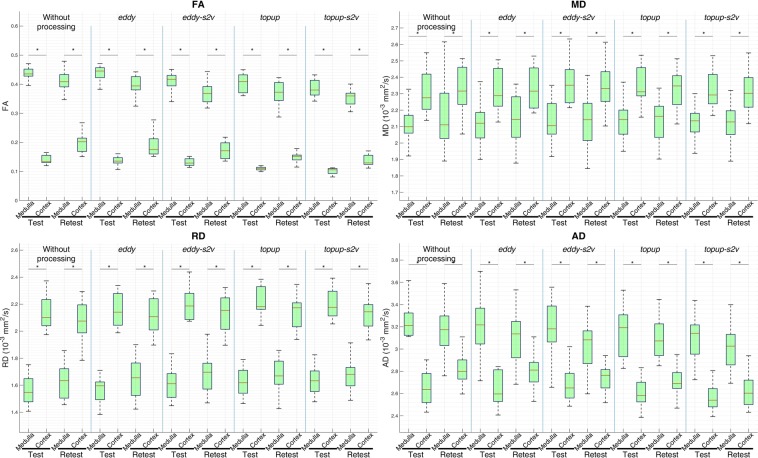
Comparison of cortical and medullary DTI parametric map values (FA: fractional anisotropy; MD: mean diffusivity; RD: radial diffusivity; AD: axial diffusivity) for each processing pipeline (without processing, *eddy*, *eddy-s2v*, *topup*, *topup-s2v*), for both test and retest data. MD, RD and AD values are expressed in 10^−3^ mm^2^/s. Red bars represent the median and green boxes include from 25th to 75th percentile of the samples. Asterisk symbol indicates statistically significant difference.

**Figure 4:**
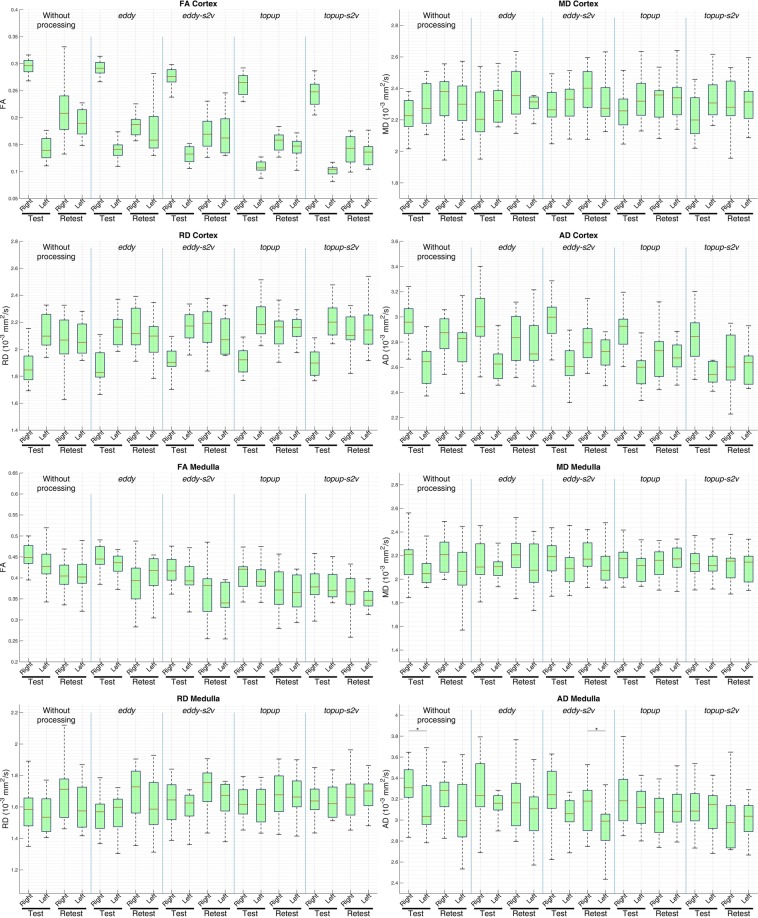
Cortical and medullary left and right kidney values in DTI parameters (FA: fractional anisotropy; MD: mean diffusivity; RD: radial diffusivity; AD: axial diffusivity) for each processing pipeline (without processing, *eddy*, *eddy-s2v*, *topup*, *topup-s2v*), for both test and retest data. MD, RD and AD values are expressed in 10^−3^ mm^2^/s. Red bars represent the median and green boxes include from 25th to 75th percentile of the samples. Asterisk symbol indicates statistically significant difference.

In Fig. 3 the average values of the parametric map from cortical and medullary regions evaluated from each processing pipeline and for both test and retest datasets are summarized. Moreover, the Fig. 4 shows the cortical and medullary left and right kidney difference in DTI parametric maps for each processing pipeline and for both test and retest datasets. Values of wsCV and ICC are summarized in Table 1. Mean wsCVs are 0.083, 0.074, 0.070, 0.055 and 0.053 for parametric maps without any image processing procedure and derived from *eddy*, *eddy-s2v*, *topup* and *topup-s2v* pipeline, respectively. Moreover, mean ICC values are 0.598, 0.680, 0.668, 0.796, 0.814 for parametric maps without processing and derived from *eddy*, *eddy-s2v*, *topup* and *topup-s2v*, respectively.

**Table 1:** Medullary and cortical within-subject coefficient of variation (wsCV) and intra-class correlation coefficient (ICC) for each processing pipelines.

	Medulla				Cortex			
	FA	MD	RD	AD	FA	MD	RD	AD
	**wsCV**							
Without processing	0.097	0.056	0.042	0.094	0.273	0.020	0.050	0.031
*eddy*	0.099	0.036	0.046	0.064	0.253	0.014	0.049	0.029
*eddy-s2v*	0.114	0.033	0.050	0.057	0.218	0.019	0.033	0.030
*topup*	0.088	0.013	**0.026**	0.040	0.229	**0.011**	0.031	0.027
*topup-s2v*	**0.076**	**0.013**	0.030	**0.028**	**0.215**	**0.011**	**0.027**	**0.023**
	**ICC**							
Without processing	0.415	0.479	0.693	0.335	0.319	0.880	0.802	0.858
*eddy*	0.372	0.721	0.759	0.519	0.410	0.951	0.755	**0.952**
*eddy-s2v*	0.337	0.734	0.706	0.596	**0.432**	0.876	0.840	0.822
*topup*	0.672	0.947	**0.940**	0.816	0.208	0.944	**0.928**	0.916
*topup-s2v*	**0.734**	**0.953**	0.931	**0.885**	0.254	**0.952**	0.878	0.922

FA indicates fractional anisotropy; MD, mean diffusivity, RD, radial diffusivity, AD, axial diffusivity. Highest values are highlighted in bold text format. Please refer to the text for the details of the processing pipelines.

### Spatial overlap

**Figure 5:**
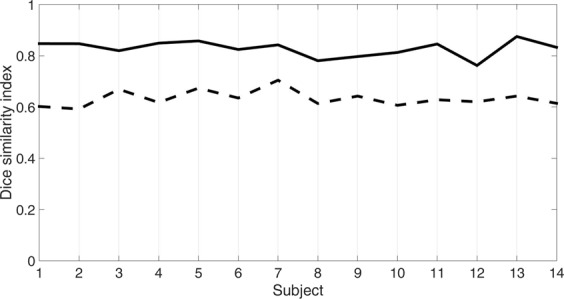
Geometric distortion correction assessment. For each participant in this study, the plot shows the Dice similarity index evaluated between the segmented kidney masks from HASTE and the b0-image without processing (dashed line) and obtained with *topup-s2v* pipeline (solid line).

**Figure 6:**
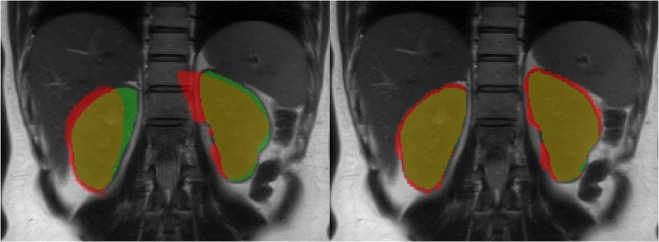
Comparison of kidney segmentation results obtained from the b0-image without processing (left) and with *topup-s2v* pipeline (right) overlaid on HASTE image. Green and red pixels correspond to the mask obtained from HASTE and b0-image, respectively. Pixels in yellow colour represent the overlap between masks from HASTE and b0-image.

Geometric distortion correction was successfully performed for all participants. The Dice similarity index obtained with b0-image without processing range from 0.59 and 0.70 (mean ± standard deviation: 0.63 ± 0.03) and from 0.76 and 0.87 (mean ± standard deviation: 0.83 ± 0.03) when *topup-s2v* pipeline was applied. In Fig. 5 the spatial overlap results in terms of Dice index are shown for each subject in this study. Moreover, in Fig. 6 an example of the kidney segmentation masks overlaid on HASTE image is displayed.

## Discussion

In this work, we show that the choice of the optimal processing pipeline in kidney DTI is critical for both standardization and reproducibility of the results. To date, a high heterogeneity of processing pipelines was found in literature^31^ and no consensus was reached on the optimal processing protocol in kidney DTI. To the best of our knowledge, this is the first study where the comparison between different processing pipelines is achieved and a reliable processing protocol in kidney DTI is proposed. In this study, five different processing pipelines are tested in order to compare the results in terms of reproducibility of DTI parametric map values.

The acquisition protocol of the presented study is designed to make the DTI data comparable with those present the literature. The coronal slice orientation of DTI images is chosen since whole kidneys can be imaged with reduced number of slices and, consequently, reduced scan time respect to axial or sagittal image acquisition strategies. However, the coronal acquisition is mainly prone to respiratory movement artefacts and it can be considered as an increased difficulty to deal with. Moreover, the respiratory triggering is adopted during the DTI acquisition since it was demonstrated the feasibility of kidney DTI with excellent cortico–medullary differentiation^17^. Therefore, we select a b-value of 500 s/mm^2^ since it can be considered as a good compromise between image quality and pure diffusion measurement^14,46^. In addition, considering that less restrictive structures require lower b-value to be revealed by diffusion imaging^47^, the choice of optimal b-value depends on water diffusion constrained by the underlying anatomical components that, in the case of the healthy kidneys are less restricitive structures (i.e. collector ducts and tubules). In this setting, we select the optimal b-value by considering the MD values of kidneys reported in literature^32,34^.

The obtained parametric map values are consistent with those presented in kidney DTI studies of healthy volunteers for similar acquisition protocols and age range^14,21,32,48–50^. Moreover, as pointed out in Fig. 3, FA and AD values are considerably higher in medulla respect to cortex and, conversely, MD and RD values are higher in cortex respect to medulla. The obtained results, confirmed for all processing pipelines, are in line with those presented in literature^15,16,33^. The radial orientation of tubules in the renal medulla produces more restricted movement of the water molecules respect to cortex and, as a consequence, FA and AD (the preferential diffusion direction and the diffusivities perpendicular to the principal diffusion direction, respectively) maps result in higher values in the medulla than the cortex. Conversely, since MD and RD parametric maps represent the mean diffusivity and the diffusivity parallel to the principal diffusion direction respectively, it is expected to observe higher values of such diffusivity parameters in the cortex respect to medulla^51^. According to the results found in literature^32,34,50^, the overall paired t-test results from right and left kidney in terms of parametric map values show no consistent difference. However, as shown in Fig. 4, medullary AD values carried out both without processing and with *eddy-s2v* considerably differ between right and left kidney. The unwanted results might be explained by the influence of an improper processing pipeline. The influence of the different processing pipelines is also visually confirmed in Fig. 1 where the image artefacts related to eddy-currents and susceptibility-induced distortions are mitigated in both *topup* (Fig. 1e) and *topup-s2v* (Fig. 1f) processing approaches.

Test-retest analysis shows a good reproducibility of the results in terms of both wsCV and ICC values (Table 1). In particular, *topup-s2v* processing pipeline demonstrates to reach best reproducibility. *Topup-s2v* also achieves best tractography results by providing both pronounced differentiation of renal pyramids and proper anatomical delineation of renal border (Fig. 2). In terms of spatial overlap between HASTE and DTI images, Dice index are increased by 0.2 on average when *topup-s2v* pipeline is applied respect to DTI images without processing, thus confirming the proper correction of susceptibility-induced geometric distortion. Moreover, the Dice indices are almost constant between the subjects of this study (Fig. 5) thus reflecting the high reproducibility of the geometric distortion correction results.

Our study has several limitations. First, the sample size is small thus reflecting a low power of the statistical analysis. The small size of our dataset is mainly due to the experimental nature of this study in which we had to ask healthy volunteers to undergo, twice, long scanning procedures without receiving a direct clinical benefit. With the assessment of a reliable processing pipeline, we are confident that shorter acquisitions, suitable for adequate sample size of patients, can be deployed for DTI-based biomarker discovery. Second, the participants are young healthy volunteers and further studies must be conducted to confirm the presented results on a sample with broader age range. Finally, the tractography reconstructions are qualitatively evaluated and a quantitative assessment of the tractography reproducibility must be provided with further studies.

In the present study the assessment of both test-retest analysis and geometric distortion correction is performed by means of manual annotation procedures. Automated segmentation routines^52^ can overcome the limitations related to the manual tracing thus increasing the reproducibility of results. In this setting, future investigations should be provided to by using an automated segmentation approach.

In addition, the proposed study evaluates the reproducibility of DTI parametric maps and further investigations can be useful to assess the results of different processing pipelines also in case of more complex diffusion models as intravoxel incoherent motion (IVIM) imaging^53^. In this context, the prolonged scan time due to the large amount of acquisitions with different diffusion weightings can result in noticeable movement artefacts and the choice of the optimal processing pipeline is non-trivial.

In conclusion, the processing procedure can dramatically affect the reliability of kidney DTI. We propose a robust processing pipeline that, including off-resonance induced geometric distortion and both volume-to-volume and slice-to-volume motion correction, ensures highly reproducible results.

## Supplementary information

Supplementary info

## Data Availability

MR data can be made available under request to the corresponding author (pending the approval of the Research Ethics Board).
