# Supplementary material for: Diffusion Tensor Imaging of the Kidney: Design and Evaluation of a Reliable Processing Pipeline
Source: Sci Rep. 2019 Sep 4;9:12789. doi: 10.1038/s41598-019-49170-5 (PMC6726597; doi:10.1038/s41598-019-49170-5)
Supplement: Supplementary file 1 — Supplementary info [file 41598_2019_49170_MOESM1_ESM.docx]

Diffusion Tensor Imaging of the Kidney: Design and Evaluation of a Reliable Processing Pipeline

Supplementary Information

Pasquale Borrelli, Carlo Cavaliere, Luca basso, Andrea Soricelli, Marco Salvatore & Marco Aiello

Supplementary Information

**Table S1:** Summary of artefact correction methodologies used for each image processing pipeline.

|  | Eddy-currents correction | Volume-to-volume movement correction | Susceptibility-induced distortion correction | Slice-to-volume movement correction |
| --- | --- | --- | --- | --- |
| Without processing |  |  |  |  |
| *eddy* | x | x |  |  |
| *eddy-s2v* | x | x |  | x |
| *topup* | x | x | x |  |
| *topup-s2v* | x | x | x | x |


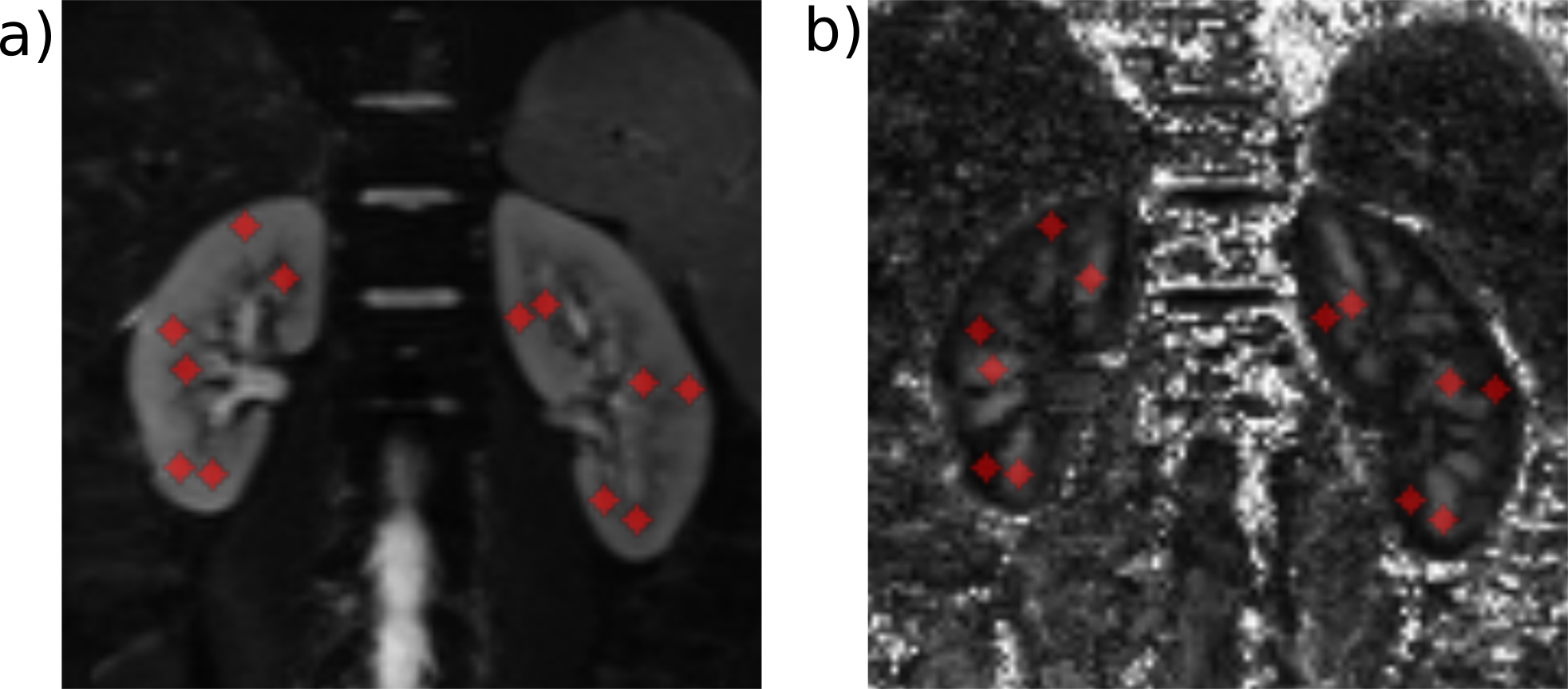


**Figure S1:** Representative location of circular regions of interest (red circles) positioned in upper, middle and inferior kidney segments overlaid both on b0-image (a) and FA map (b) obtained with *topup-s2v* processing pipeline (please refer to the text for the details of *topup-s2v* processing pipeline).

**
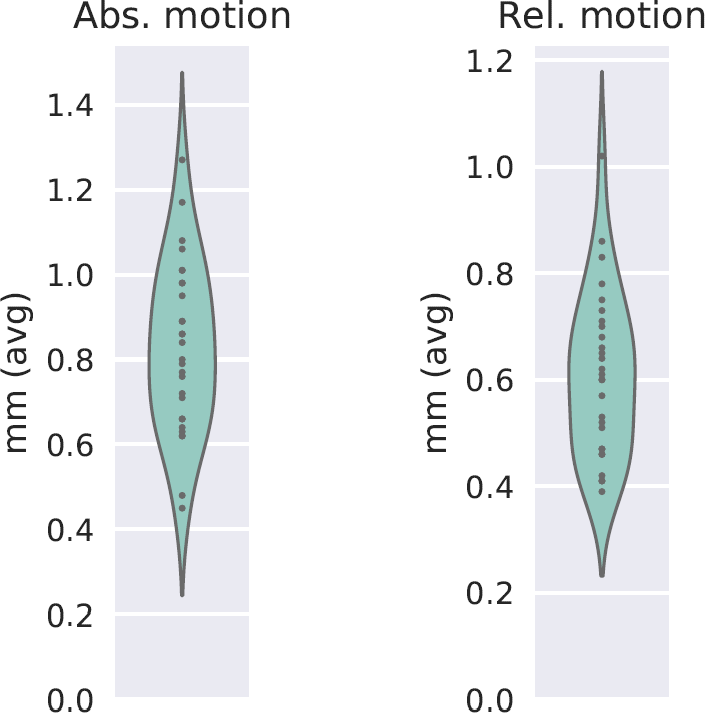
**

**Figure S2:** Summary of absolute (left) and relative (right) movement in mm calculated with an automatic quality control routine. The absolute and relative motion estimates are represented as averages over all volumes of each diffusion tensor imaging data. Each grey dot in the violin plots represents one of 14 participants (separately for test and retest), thus showing 28 different points. Please consider that there are 6 overlapping points in the violin plot related to absolute movement and 4 overlapping points for the violin plot of the relative motion.
